# Bilateral Simultaneous Nonarteritic Anterior Ischemic Optic Neuropathy after Ingestion of Sildenafil for Erectile Dysfunction

**DOI:** 10.1155/2012/747658

**Published:** 2012-03-19

**Authors:** Anna Tarantini, Alessandra Faraoni, Francesca Menchini, Paolo Lanzetta

**Affiliations:** Department of Ophthalmology, University of Udine, 33100 Udine, Italy

## Abstract

*Purpose*. To describe a patient who developed bilateral, simultaneous nonarteritic anterior ischemic optic neuropathy (NAION) after ingestion of Sildenafil citrate (Viagra) for erectile dysfunction. *Methods*. Observational case report. *Results*. A 60-year-old diabetic man noted sudden decrease of vision in both eyes 16 hours after his third consecutive 50 mg daily Sildenafil ingestion. A diagnosis of bilateral NAION was made and he was treated for three days with methylprednisolone 1 g/d intravenously, followed by oral prednisone 75 mg/d. Final visual acuity was 20/50 right eye (OD) and 20/20 left eye (OS). He had preexisting diabetes. *Conclusion*. This is the first reported case of simultaneous bilateral NAION occurred in a diabetic patient early after Sildenafil intake. Patients with predisposing conditions such as diabetes have to be warned against the use of PDE inhibitors.

## 1. Introduction

Sildenafil citrate (Viagra, Pfizer Pharmaceuticals, New York, NY) inhibits selectively cyclic guanosine monophosphate (cGMP) specific phosphodiesterase type 5 (PDE 5) and is used to treat erectile dysfunction. Sildenafil is able, by enhancing the effect of nitric oxide and cGMP pathway, to lead to smooth muscle relaxation in the corpus cavernosum, allowing inflow of blood during sexual stimulation. PDE-5 inhibitors have been reported to cause transient changes in colour perception (objects have blue or blue-green tinges) or changes in lightness perception (usually an increased sensitivity), blurred vision, and transitory ERG changes [1]. Possible additional ocular side effects associated with Sildenafil or other PDE 5 inhibitors such as Vardenafil and Tadalafil use are mydriasis, retinal vascular accidents, conjunctival hyperemia, ocular pain, subconjunctival hemorrhage, and ischemic optic neuropathy [1]. With regard to severe ocular adverse effects, PDE-5-inhibitors-associated nonarteritic anterior ischemic optic neuropathy (NAION) has been reported in 49 subjects. Forty-four are related with the use of Sildenafil, 4 with the use of Tadalafil, and 1 with the use of Vardenafil [2–7]. Few bilateral sequential cases have been reported. More recently, Moschos and Margetis described a single case with bilateral simultaneous anterior ischaemic optic neuropathy which occurred in a man with unremarkable medical history 8 months after continuous use of Sildenafil [7].

The purpose of the present case report is to describe a diabetic patient who developed a simultaneous bilateral NAION early after ingestion of Sildenafil for erectile dysfunction.

## 2. Case Report

A 60-year-old diabetic man took one 50 mg tablet of Sildenafil in the evening for 2 consecutive days without any effects and he was unable to have intercourse. On the third day he discontinued antiglycaemic medications, took another 50 mg tablet and engaged in sexual activity. Sixteen hours later he noted sudden decrease of vision in both eyes with a pronounced worsening in the right eye. He was admitted in a nearby hospital where a CT brain and a chest radiography were performed. They were unremarkable as well as complete blood count and the erythrocyte sedimentation rate. His medical history was significant for noninsulin-dependent diabetes for 7 months and his medication was metformin. He was discharged from hospital with a new prescription of 100 mg daily aspirin.

**Figure 1:**
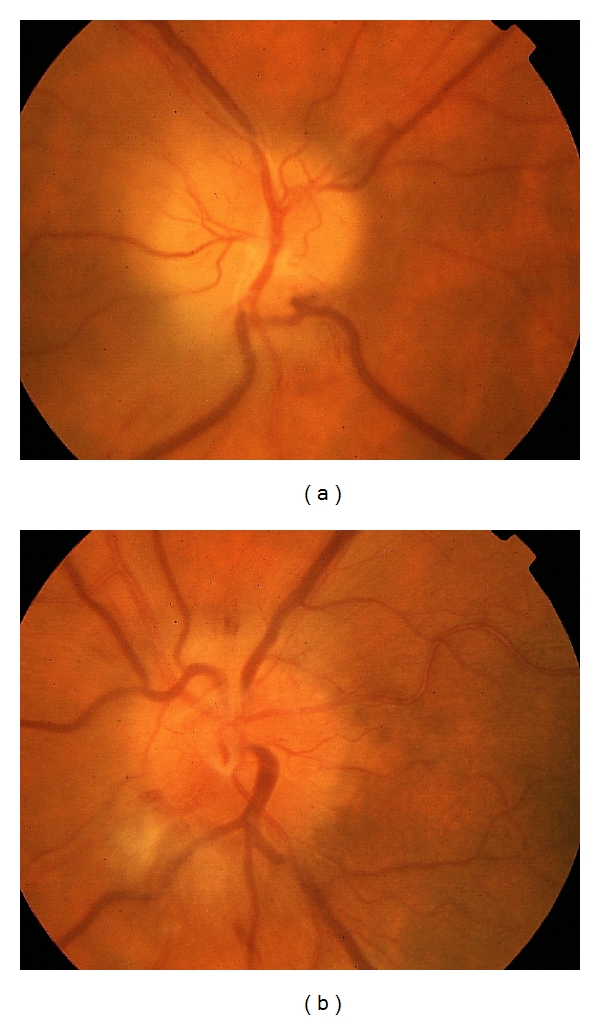
Color fundus photo of OD (a) and OS (b) with optic disc edema and peripapillary nerve fiber layer hemorrhages (a, b) and peripapillary cotton wool spots (b).

**Figure 2:**
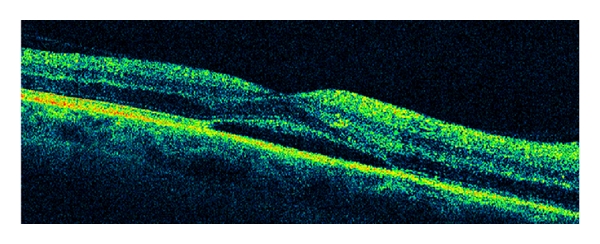
Optical coherence tomography shows subretinal fluid in the macula in OD.

**Figure 3:**
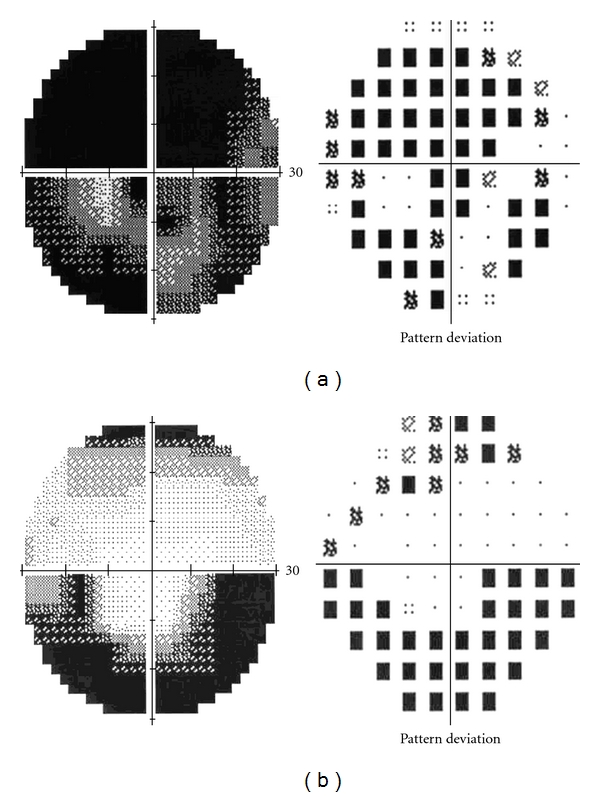
Test results of Humphrey visual field. Superior altitudinal and central defects OD (a) and inferior altitudinal defect OS (b).

**Figure 4:**
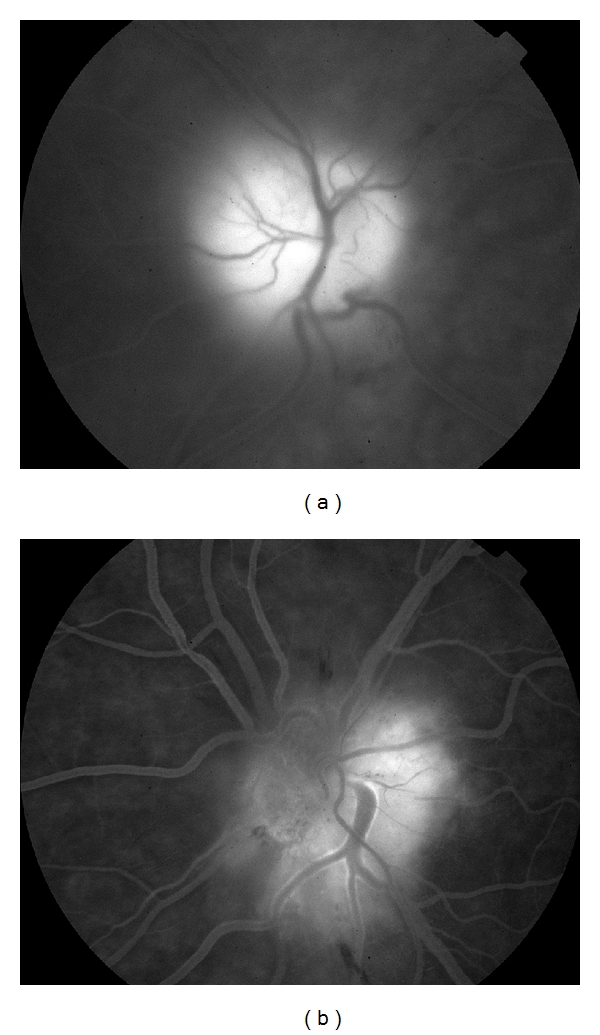
Fluorescein angiography shows late dye leakage in both eyes (a, b).

Seven days after the onset of symptoms, the patient was hospitalized at our department. Visual acuity was 20/63 right eye (OD) and 20/32 left eye (OS). On fundus examination, optic disc edema and peripapillary nerve fiber layer hemorrhages were disclosed in both eyes (Figure 1). Serous macular detachment was present in OD (Figure 2) whereas peripapillary cotton wool spots were found in OS. No evidence of diabetic retinopathy was noted. Blood pressure was within normal limit during the admission period. Humphrey visual field testing showed superior altitudinal and central defects OD and inferior altitudinal defect OS (Figure 3). A fluorescein angiogram showed late leakage in the optic disc of both eyes (Figure 4). Symptoms of giant cell arteritis were not present and no relative afferent pupillary defect was detected. A diagnosis of bilateral NAION was made and he was treated for three days with methylprednisolone 1 g/d intravenously, followed by oral prednisone 75 mg/d. The prednisone dose was tapered and discontinued over one month. Two weeks after the last steroid i.v. administration, visual acuity had increased to 20/50 OD and 20/20 OS. Optic disc edema, sub-retinal fluid and serous macular detachment resolved in OD and optic disc edema improved in OS. No sign of dye leakage of the optic disc was found in eyes and visual field testing disclosed altitudinal defects in both eyes. Three months later, visual acuity was stable and the optic disc was pale in OD.

## 3. Discussion

The incidence of bilateral AION reported in the general population varies from 10.5% to 73% [8]. This wide variation is due to a number of factors such as the retrospective retrieval of data in almost all studies and the length and quality of followup. In the Ischemic Optic Neuropathy Decompression Trial (IONDT) Followup Study [9], new NAION in the fellow eye occurred in 14.7% of patients at risk during a median followup of 5.1 years. In a prospective study by Beri et al. [8], the estimated 25th-percentile time to bilaterality from the onset of AION in the first eye in NAION patients was 32.4 months and it decreased to 17.6 months in the group matched with our patient by sex and age (45–64 years) and to 9.8 months in the diabetes group. Bilateral simultaneous anterior ischemic optic neuropathy is rare. Shibayama et al. [10] reported a case of bilateral and nearly simultaneously occurring NAION in a 61-year-old man. The patient had two of the established risk factors for NAION such as a poorly controlled diabetes and a small disc with a small physiologic cup. Bilateral simultaneous NAION after Sildenafil has been previously reported in a 55-year-old man with an unremarkable medical history 8 months after continuous use of Sildenafil 4-5 times a month [7]. The authors did not disclose whether the patient developed bilateral simultaneous NAION soon after ingesting the drug.

Sexual stimulation triggers the release of nitric oxide (NO) from noncholinergic, nonadrenergic nerve endings and from endothelial cells of arteries and sinusoids of corpus cavernosum. NO activates guanylyl cyclase to increase intracellular cGMP production that produces relaxation of the corpus cavernosum and penile arteriolar smooth muscle determining a subsequent erection. Sildenafil inhibits phosphodiesterase type 5 and this results in higher concentrations of cGMP that activates cGMP-dependent protein kinase (PKG). 

Studies on the ocular circulation in healthy subjects support the involvement of endogenous NO derived from either endothelial cells or perivascular nitrergic neurons in the control of vascular smooth muscle tone. A sufficient blood supply in the ocular circulation requires the maintenance of a basal vasodilator tone in ocular arteries. Like in many other arterial beds, this is provided by a constant formation of NO. A dysbalance of the NO-system contributes to alterations in systemic vascular diseases like hypertension or diabetes, where basal formation of NO as well as NO dependent vasodilatation is impaired.

Human and animal studies suggest that alterations of the NO system in part mediate blood flow changes in states of hyperglycemia. High glucose (500 mg/dL) significantly decreased nitrite production compared with normal glucose (100 mg/dL) whereas PKG activator treatment induced high level of NO, inducible nitric oxide synthase and PKG in high glucose-incubated cells [11]. Therefore, a situation of hyperglycemia and PKG activation may be characterized by an enhancement of the NO-pathway. It is noteworthy to mention that our patient was able to have intercourse only on the third day after he took Sildenafil and avoided his oral medications for diabetes with consequent presumed secondary hyperglycemia. It is also interesting to underline that not only concentrations of NO are elevated in the vitreous of patients with proliferative diabetic retinopathy but one of the nitric oxide synthase (NOS) byproducts is increased in aqueous humor of diabetic patients with or without diabetic retinopathy indicating that NOS activity is already increased in early stages of insulin resistance such as in type 2 diabetes mellitus. NO as well as hypoxia is reported to upregulate the vascular endothelial growth factor (VEGF) gene, although there are some reports on the negative effects of NO on VEGF activity. These conflicting data of NO effects may be attributed mainly to the amount of released NO. Indeed, NO can be a positive or negative modulator of the VEGF gene under the same conditions simply by changing its amounts [12]. NO is capable of stimulating angiogenesis and vascular permeability via interplay with VEGF. Since intravenous administration of VEGF to experimental animals increased endothelial permeability and blood flow in retina, choroid, and anterior uvea, its acute expression may result in damaging edema and secondary injury during NAION.

Phosphodiesterase 5 inhibitors, in the cardiovascular system, reduce arterial systemic blood pressure and, as regarding the hemodynamic effect on ocular circulation, it is known that Sildenafil significantly increases, rather than decreases, blood flow velocity in the retrobulbar and choroidal circulation. Other mechanisms have to be further investigated. In patients with a predisposing diabetic condition, Sildenafil intake can cause changes in NO balance altering the normal vascular autoregulation so that the ocular circulation may not be able to compensate for a drop in systemic blood pressure. Moreover, the indirect involvement of Sildenafil in the NO system with effects on inducible NO synthase and the VEGF signaling pathway may lead to leukocyte vascular adhesion, downregulation of tight junction protein, and breakdown of the blood-ocular barrier leading to NAION damages.

A new class of hydrogen sulfide (H(2)S)-donating hybrids combined with sildenafil might show improved tolerability with potential cytoprotective effect of H(2)S on retinal neurons [13, 14].

Our patient presented not only with bilateral NAION but also with serous macular detachment in the OD that has been previously reported [15] due to the vasodilator action of Sildenafil that causes an idiosyncratic engorgement of the choroidal vasculature leading to leakage across the retinal pigment epithelium and accumulation of subretinal fluid.

This is the second reported case of bilateral simultaneous NAION in a Sildenafil consumer. Differently from the case described by Moschos and Margetis [7], our case is characterized by the sporadic use of Sildenafil, the occurrence of the neuropathy early after Sildenafil ingestion, and the association with diabetes which is a recognized risk factor for vascular accidents.

In conclusion we believe that general practitioners and neuroophthalmologists should recommend against the use of PDE inhibitors not only in patients who have already experienced an episode of NAION in one eye, as stated by the European Supplementary Protection Certificate class labelling, or have a small disc-at-risk, but also in patients with predisposing systemic vascular diseases like hypertension or diabetes.

A phase IV observational case-crossover study to assess whether PDE 5 inhibitors increase the chance of triggering the onset of acute NAION is currently ongoing (http://www.clinicaltrials.gov/ct2/show/NCT00759174?term=naion&rank=1), and the results are expected to be available in the near future and will provide useful data on the safety of these compounds and on patients at risk who should avoid their use.
